# Lipids of *Quercus* Acorns: Health Implications, Oxidative Stability, and Food Applications

**DOI:** 10.3390/foods15152747

**Published:** 2026-08-05

**Authors:** Petra Lončarić, Marko Jukić, Tihomir Moslavac, Jasmina Lukinac

**Affiliations:** Faculty of Food Technology Osijek, Josip Juraj Strossmayer University of Osijek, F. Kuhaca 18, 31000 Osijek, Croatia; ploncaric@ptfos.hr (P.L.); marko.jukic@ptfos.hr (M.J.); tihomir.moslavac@ptfos.hr (T.M.)

**Keywords:** *Quercus*, fatty acid composition, oleic acid, tocopherols, phytosterols, bioactive compounds, starch–lipid interaction, functional food

## Abstract

Acorns (*Quercus* spp.) have re-emerged as a sustainable, underutilized food resource, and their lipid fraction has attracted growing scientific interest because of its distinctive composition and technological potential. Acorn lipids typically represent 5–12% of acorn dry weight, ranging from 0.75% (*Q. vulcanica*) to over 17% (*Q. rubra*), and are dominated by unsaturated fatty acids, mainly oleic acid (up to ~70% of total fatty acids) and, to a lesser extent, linoleic acid, alongside minor bioactive compounds such as tocopherols (31.60–1486 mg/kg oil), phytosterols (2449–8797.92 mg/kg oil), carotenoids (β-carotene: 473–1421 mg/kg DM), and long-chain aliphatic alcohols (2198.81–2248.87 mg/kg oil) associated with antioxidant, anti-inflammatory, hypocholesterolemic, and cardioprotective effects. Despite their high degree of unsaturation, acorn oils maintain good oxidative stability during up to 180 days of storage under dark conditions at room temperature, partly due to these endogenous antioxidants, and their lipids participate in starch–lipid interactions that influence starch digestibility, glycemic response, and the functional properties of acorn-based foods, including bakery, gluten-free, and pasta products. Based on a comprehensive literature search of the Web of Science database through June 2026, this review provides a comprehensive synthesis of acorn lipids. Critical analysis shows that most evidence derives from whole acorn flour rather than isolated lipids, that reporting units remain inconsistent across studies, and that dedicated intervention trials using acorn oil are lacking—gaps that should guide future research. This review serves as a critical bridge between botanical lipidomics and food engineering, providing a framework for the standardized valorization of *Quercus* species in the global transition to sustainable, plant-based functional ingredients.

## 1. Introduction

Acorns are the fruits of trees of the genus *Quercus* and have been part of human nutrition since prehistoric times, once representing an important dietary component in many Mediterranean, Asian, and North American regions. Archaeobotanical evidence and historical records indicate that acorns were processed into flour, bread, porridge, beverages and edible oil, particularly in regions where cereal production was limited or during periods of food scarcity [1,2,3]. Their importance as a food source declined with the development of modern agriculture and the increasing availability of cultivated cereals, after which acorns were mainly relegated to animal feed or emergency food despite their favorable nutritional properties [4]. In recent years, however, interest in sustainable foods, dietary variety and the valorization of underutilized plant resources has grown. Their natural abundance, adaptability to diverse ecosystems, and suitability for low-input production make them an attractive raw material aligned with current strategies for improving food security and promoting more sustainable food production [4,5,6,7]. Consequently, scientific research has progressively shifted from documenting the historical significance of acorns to characterizing their nutritional composition, bioactive compounds, and technological potential for functional foods.

The nutritional composition of acorns varies across *Quercus* species and is influenced by geographical origin, climatic conditions and stage of maturity. Acorns contain starch, dietary fiber, proteins, minerals and lipids, together with numerous bioactive phytochemicals [8,9,10]. Among these components, the lipid fraction is of particular interest because of its high proportion of unsaturated fatty acids and the presence of minor bioactive lipid compounds that contribute to the nutritional and functional properties of acorn-derived foods [5,8,11].

Carbohydrates (mainly starch) are the major fraction of acorn flour and dictate many of its technological characteristics, including gelatinization behavior and digestibility [12]. However, acorns also contain significant amounts of minerals, tocopherols, phytosterols, carotenoids and other bioactive compounds that have been associated with antioxidant activity and other health-promoting properties. Although the lipid fraction generally ranges only from approximately 5 to 12% of acorn dry weight, it is characterized by a high proportion of unsaturated fatty acids (UFAs) and several minor bioactive constituents. This composition distinguishes acorn lipids from many other forest-derived foods and suggests that they contribute to the nutritional and technological properties of acorn-enriched products [5,13,14,15].

The lipid fraction of acorn flour has gained increasing scientific interest because of its distinctive composition and potential health benefits. Compared with many other edible nuts and cereal grains, acorn lipids are characterized by a predominance of monounsaturated fatty acids (MUFAs), particularly oleic acid, followed by lower proportions of linoleic, palmitic, stearic and α-linolenic acids [13,16,17,18]. Acorn oil also contains several minor lipid compounds with bioactive properties: tocopherols, phytosterols, carotenoids and long-chain aliphatic alcohols. These minor lipid compounds have been associated with antioxidant, anti-inflammatory and cardioprotective activities [11,14,15].

Beyond their nutritional relevance, acorn lipids are also technologically significant. They participate in starch–lipid interactions that influence starch digestibility and glycemic response, contribute to oxidative stability through their endogenous antioxidant content, and affect the functional properties of acorn-based foods, including dough rheology, texture, and shelf life [19,20].

Although the number of published studies on this topic has increased, research on acorn lipids remains fragmented. Most investigations have focused on the nutritional composition or functional properties of whole acorn flour. This makes it difficult to distinguish the specific contribution of the lipid fraction from that of starch, dietary fiber, proteins and phenolic compounds. In addition, there is considerable diversity in the analytical methods, units of expression and reporting of lipid composition among published studies, which complicates direct comparisons between *Quercus* species and limits the comprehensive interpretation of available data. Moreover, while the nutritional composition of acorn lipids has been relatively well described, their specific role in food processing and interactions with other food components remains insufficiently understood. To date, relatively few reviews have focused specifically on acorn lipids as a stand-alone subject, despite their nutritional and technological importance.

The aim of this review is to summarize and critically evaluate the available literature on the lipid fraction of acorns, with particular attention to lipid composition, minor bioactive compounds, species- and geography-related variability, health implications, oxidative stability, and technological applications in food systems. This review seeks to present current knowledge on acorn lipids in a consistent and thorough manner, identify existing research gaps, and provide direction for future investigations in this field.

## 2. Materials and Methods

### 2.1. Literature Search Strategy

A literature search was conducted to find studies about the composition, nutritional significance, oxidative stability and food applications of acorn lipids. The primary database used for the literature search was Web of Science and was sometimes supplemented by additional searches of reference lists from relevant publications.

The search was conducted between March and June 2026 and included publications available up to June 2026. Keywords were adapted according to the topic of each section and included combinations of the following terms: “acorn lipids”, “acorn oil”, “*Quercus*”, “fatty acids”, “oleic acid”, “linoleic acid”, “unsaturated fatty acids”, “tocopherols”, “phytosterols”, “long-chain aliphatic alcohols”, “carotenoids”, “oxidative stability”, “lipid oxidation”, “health benefits”, “acorn flour”, “acorn bread”, “gluten-free products”, “acorn bakery products”, “food applications”, “storage stability” and “shelf life”.

### 2.2. Study Selection

Studies were considered suitable if they contained original data or review information on the lipid fraction of acorns, including lipid content, fatty acid composition, minor bioactive lipid compounds, oxidative stability, nutritional significance, or functional food applications. Research investigating different *Quercus* species, geographical origins and acorn-enriched food products was also included.

Studies focusing exclusively on non-food applications of acorns and studies without sufficient information relevant to acorn lipids were excluded.

### 2.3. Data Extraction and Synthesis

Relevant information was extracted from the selected studies and organized according to the aims of the review. Data about lipid content, fatty acid composition, tocopherols, phytosterols, carotenoids, oxidative stability parameters, health-related properties and food applications were collected and compared across studies. Particular attention was devoted to differences among *Quercus* species and geographical origins. The collected literature was used to provide a comprehensive overview of the current state of knowledge on acorn lipids and to identify existing knowledge gaps.

## 3. Lipid Composition of Acorns

Acorns represent a chemically variable raw material, and this variability is important to consider when evaluating and interpreting their lipid composition. In general, acorns contain considerable amounts of starch, dietary fiber, minerals, phenolic compounds with antioxidant activity and lipids rich in UFAs [12,21,22].

Differences among *Quercus* species extend beyond morphology and geographic distribution to major nutritional components, including carbohydrates, particularly starch, proteins, minerals, lipids and bioactive compounds [16,23,24]. Since the chemical composition of seeds is strongly influenced by genetic factors, acorn composition may differ considerably among species, varieties and populations [16,25]. In addition, environmental conditions, stage of maturity, geographic origin and processing or extraction methods may further contribute to differences in reported values [26,27,28]. Therefore, lipid composition should not be interpreted as a stable characteristic of acorns, but as a variable feature that is influenced by both biological and external factors. This section focuses on the main aspects of acorn lipid composition, including total lipid content, fatty acid profile and minor bioactive lipid components.

### 3.1. Variability in Lipid Content Among Acorn Species

Considerable variability in the general chemical composition of acorn species also extends to their lipid content [25]. The lipid content of acorns varies considerably among species and sources. Kowalska et al. (2026) [17] reported that the lipid content of acorns, as well as their fatty acid composition, depends primarily on species.

Numerous studies have investigated the chemical composition of acorns and reported data on lipid content [5,13,16,17,18,19,23,24,29,30,31,32,33,34]. Data on geographic origin, sample matrix, total lipid content, and dominant fatty acid composition of acorns from selected *Quercus* species are compiled in Table 1, which shows that acorn lipid content ranges from 0.75% (*Q. vulcanica*) to 17.56% (*Q. rubra*) [16,17].

Several trends emerge from the available data on acorn lipid content. Among the species investigated, high lipid contents were frequently reported for Mediterranean evergreen oaks such as Portuguese *Q. ilex* with reported lipid levels of 13.41% [31] and 12.01% [29], Tunisian *Q. ilex,* with a reported lipid level of 9.32% [18] and *Q. rotundifolia* from Portugal and Spain, with lipid contents ranging from 8.44% to 12.10% [5,13,30]. However, Q. rubra from Poland had the highest lipid level of 17.56% [17]. These values exceed 10%, suggesting a greater capacity for lipid accumulation compared with many deciduous oak species. By contrast, the lowest lipid contents were reported for several species originating from Turkey (*Q. vulcanica* = 0.75%, *Q. ilex* = 0.80%, *Q. robur* = 0.99%, *Q. hartwissiana* = 1.01%, *Q. frainetto* = 1.17%, *Q. pubescens* = 1.16%) [16] and Jordan (*Q. ithaburensis* = 0.76%) [23]). These findings suggest that species is an important source of variation, although methodology and geographic origin may also strongly influence the reported lipid contents of acorns.

Reported lipid contents also vary substantially according to geographic origin. Examples include *Q. ilex* samples from Turkey, Portugal and Tunisia, with lipid contents ranging from 0.80% to 13.41% [16,18,29,31,34], *Q. robur* samples from Turkey, Portugal, Serbia and Poland ranging from 0.99% to 7.01% [16,17,30,32,34] and *Q. rubra* samples from Poland ranging from 3.09% [33] to 17.56% [17]. Even within the same country, almost a sixfold difference was observed, indicating that lipid content cannot be considered a constant species characteristic. This variation strongly supports the idea that environmental factors and/or analytical differences contribute substantially to lipid variability. These examples also identify *Q. ilex*, *Q. robur* and *Q. rubra* as the species showing the greatest variation in reported lipid content.

Table 1 also shows that both high and low lipid contents have been reported in flour and kernel samples [5,16,17,33]. Therefore, based on the available data, species identity and geographic origin may contribute more strongly to lipid variability than the analyzed matrix.

Although lipid content ranged from 0.75% to 17.56%, most reported values were between 2% and 8%, indicating that most acorn species are characterized by a moderate lipid content.

### 3.2. Fatty Acid Profile

Despite interspecific differences, most *Quercus* species exhibit a similar fatty acid profile. Generally, acorn lipids are abundant in UFAs which often represent 75–90% of total fatty acids, particularly oleic, linoleic and α-linolenic acids, while palmitic acid is generally the predominant saturated fatty acid (SFA). Oleic acid is generally the predominant fatty acid, followed by linoleic acid (n-6) and smaller amounts of α-linolenic acid (n-3) [35,36]. However, the fatty acid profiles of different acorn species exhibit considerable variation in the proportions of MUFAs, polyunsaturated fatty acids (PUFAs) and SFAs. The observed variation is generally attributed to genetic factors, species-specific characteristics, abiotic factors (e.g., degree of maturation), geographic origin and climatic conditions [28]. These factors may influence the activity of key enzymes involved in fatty acid biosynthesis and desaturation, thereby altering the relative proportions of oleic, linoleic and other fatty acids accumulated during acorn development.

Oleic acid is the most abundant fatty acid in the majority of species listed in Table 1. Its values range from 15.3% (*Q. ilex*, Turkey) to 69.37% (*Q. rotundifolia*, Spain) [16,30], while most species contain approximately 45–70% oleic acid. Particularly high oleic acid levels are consistently reported for several *Quercus* species: *Q. ilex* (59.85–69.05%) [18,24,31], *Q. rotundifolia* (60.92–69.37%) [5,13,30,31], *Q. suber* (54.59–66.44%) [18,24,29] and *Q. rubra* (64.22%) [17]. By contrast, several Turkish species show markedly lower oleic acid contents: *Q. ilex* (15.3%), *Q. pubescens* (16.9%), *Q. hartwissiana* (21.4%), *Q. robur* (22.6%) and *Q. frainetto* (25.7%) [16]. Furthermore, considerable intraspecific variation can also be observed, e.g., *Q. ilex* ranging from 15.3 to 69.05% and *Q. robur* ranging from 22.6 to 52.08%. Overall, oleic acid appears to be the characteristic fatty acid of acorns and constitutes the largest proportion of total fatty acids in most species.

Linoleic acid is the second most abundant fatty acid in nearly all species as its values range from 13.34% (*Q. ilex*, Portugal) to 49.1% (*Q. hartwissiana*, Turkey). [16,29] Most species contain approximately 15–35% linoleic acid (Table 1). The highest levels were reported for *Q. hartwissiana* (49.1%), *Q. robur* from Turkey (41.9%), *Q. frainetto* (38.7%), *Q. pubescens* (38.7%) and *Q. pontica* (35.9%) by Özcan (2007) [16]. A clear pattern can be observed in Table 1: samples with high oleic acid contents tend to have lower linoleic acid contents and vice versa. For example, *Q. rotundifolia* examined by Rodrigues et al. (2026) [30] contained 69.37% oleic acid and 17.82% linoleic acid, while *Q. hartwissiana* examined by Özcan (2007) [16] exhibited 21.4% oleic acid and 49.1% linoleic acid.

Palmitic acid is consistently the major SFA in acorns. The values range from 9.85% (*Q. rubra*) to 27.7% (*Q. ilex*, Turkey) [16,17], and most species contain approximately 12–20% palmitic acid. The highest values were observed for *Q. ilex* from Turkey (27.7%), *Q. pubescens* (22.8%), *Q. frainetto* (20.9%) [16] and *Q. faginea* (18.0%) [24]. The lowest values were reported for *Q. rubra* (9.85%) [17] and *Q. rotundifolia* from Spain (10.33%). Unlike oleic and linoleic acids, palmitic acid shows a narrower range of variation.

Other minor fatty acids occur across *Quercus* species: stearic acid is present in all species but usually accounts for less than 5% and α-linolenic acid generally ranges from 0.27 to 8.5% (Table 1). The highest α-linolenic acid contents occur in the Turkish species *Q. ilex* (8.5%), *Q. pubescens* (6.1%), *Q. pontica* (5.5%) and *Q. hartwissiana* (5.3%) [16]. Eicosenoic, vaccenic, elaidic and palmitoleic acids occur sporadically and in small amounts.

As with total lipid content, fatty acid composition is strongly influenced by geographic origin (Table 1). For example, *Q. ilex* from Turkey displayed considerably lower oleic acid and higher palmitic acid contents than samples originating from Portugal and Tunisia [16,18,24,29,31]. Similar trends can be observed for *Q. robur*, for which Turkish samples contained lower oleic acid and higher linoleic acid levels compared with Portuguese and Polish populations [16,17,30]. These observations indicate that environmental and climatic factors may contribute to the variability of acorn fatty acid composition. The pronounced divergence in oleic and linoleic acid ratios observed in Turkish accessions, compared with Iberian populations, may reflect regional environmental conditions and/or genetic differences, warranting targeted common-garden investigations. Moreover, substantial intraspecific variation, particularly in *Q. ilex* and *Q. robur*, further supports the influence of geographic and environmental factors on fatty acid composition [16,17,18,24,29,30,31].

The predominance of oleic acid, together with the varying proportions of linoleic and palmitic acids, directly influences the relative abundance of MUFAs and PUFAs in acorns. Consequently, the MUFA/PUFA/SFA balance differs among species and populations and represents an important aspect of acorn lipid quality.

Acorn oils are dominated by UFAs [17,18,34]. Haddada et al. (2025) [18] reported UFA values of 80.89–84.99%, while SFA content was much lower (15.01–19.12%) in Tunisian *Q. suber*, *Q. canariensis* and *Q. ilex* oils. Furthermore, MUFAs generally exceed PUFAs. Haddada et al. (2025) [18] reported that MUFA content ranged from 56.30% to 66.01%, whereas PUFA ranged from 16.15% to 25.99%. This shows that the unsaturated fraction is mainly represented by MUFAs, especially oleic acid. As stated earlier, species differences are clear: for example, Tunisian *Q. suber* showed the highest UFA/SFA ratio, while *Q. ilex* showed lower values; Tunisian *Q. suber* had the highest MUFA values, while *Q. canariensis* had the highest PUFA values because of its higher linoleic acid content [18]. These findings are also consistent with the fatty acid profiles reported by other authors, particularly the predominance of oleic acid over linoleic and SFAs in several *Quercus* species [5,13,17,24,29,30,31].

Processing/dehulling conditions may affect total fat and fatty acid contents, but not necessarily the lipid profile. In the study by Castro et al. (2022) [34], lipid profiles remained unaltered after thermal and drying dehulling of acorns, although fat and fatty acid contents differed depending on the dehulling method and species.

Although most studies have focused on the overall fatty acid composition of acorns, limited information is available regarding the organization of these fatty acids within different lipid classes. Lopes and Bernardo-Gil (2005) [13] reported that triacylglycerols constitute the predominant lipid class of acorn oil. Specifically, triolein represented the major molecular species (33.0–38.4%), followed by palmitoyl-diolein (12.6–18.2%) and lower amounts of other triacylglycerols. The same study also identified phosphatidylethanolamine and phosphatidylcholine as the principal phospholipids in hexane-extracted acorn oils, while supercritical CO_2_ extraction did not recover phospholipids. However, detailed characterization of lipid classes and molecular lipid species remains scarce, highlighting an important area for future research.

Taken together, the data compiled in Table 1 show that acorn lipid content and fatty acid composition are shaped simultaneously by genetic, environmental, and methodological factors, and the current literature does not allow these sources of variability to be clearly separated. Almost all available studies report single-site, single-season observations of a limited number of accessions per species, so apparent “species effects”—such as the markedly lower oleic acid and higher palmitic and linoleic acid contents reported for Turkish accessions compared with Iberian and Tunisian populations of the same or related species—may equally reflect site-specific climatic and edaphic conditions, tree age, harvest maturity, or differences in extraction and quantification procedures between laboratories. Common-garden or multi-site sampling designs that would allow the genetic and environmental components of variability to be disentangled are essentially absent from the literature. This methodological heterogeneity, together with inconsistent reporting of lipid content (dry weight versus fresh weight versus flour basis) and fatty acid data (percentage of total fatty acids versus g/100 g oil), limits the comparability of published values and constrains the reliability of any meta-analytic synthesis of acorn lipid composition. Future studies should therefore adopt standardized extraction and analytical protocols and, where feasible, common-garden or multi-origin sampling designs to determine the relative contributions of genotype, environment, and analytical methodology to the observed variability.

### 3.3. Minor Bioactive Lipid Components

Tocopherols are lipophilic bioactive compounds and natural antioxidants [37]. They represent the most frequently reported minor lipid components in acorns as they were reported in almost all species included in Table 2. Nevertheless, tocopherol concentrations show substantial variability, ranging from approximately 31–45 mg/kg in *Q. coccifera, Q. faginea*, *Q. ilex*, *Q. pyrenaica* and *Q. suber* [38] to 1006–1486 mg/kg in *Q. rotundifolia* and *Q. suber* [13], with *Q. suber* generally exhibiting relatively high tocopherol concentrations [13,38,39]. Most studies identified α-, γ- and δ-tocopherols, while some also reported β-tocopherol and α-tocotrienol. A clear pattern is that γ-tocopherol predominates in most species, including *Q. coccifera*, *Q. ilex*, *Q. pyrenaica*, *Q. suber*, *Q. robur* and *Q. rotundifolia*; however, its concentration varied among studies of the same species, which may reflect differences in geographic origin, environmental conditions and analytical methods used. Furthermore, exceptions exist: *Q. canariensis* was characterized mainly by α-tocopherol [15]; *Q. rubra* showed β-tocopherol predominance [28]; and *Q. faginea* showed α-tocopherol predominance in Vinha et al. (2016) [24], whereas Akcan et al. (2017) [38] found γ-tocopherol predominance in the same species.

Phytosterol composition is quite consistent in acorns, with β-sitosterol being the predominant compound. Its concentration exceeds 90% of total sterols in *Q. coccifera* and *Q. ilex* [40], while campesterol and stigmasterol are repeatedly reported as secondary sterols. This suggests that phytosterol composition may be more conserved across *Quercus* species than fatty acid composition (Table 1). Total sterol concentrations vary considerably among species, ranging from approximately 2449 mg/kg oil (*Q. robur*) to nearly 8800 mg/kg oil (*Q. suber* and *Q. ilex*). Particularly high sterol concentrations were reported in Mediterranean species such as *Q. ilex* and *Q. suber*. This observation may resemble the trend previously observed for lipid content and oleic acid abundance.

Carotenoids and long-chain aliphatic alcohols are minor lipid components that are less frequently reported. β-Carotene consistently exceeds lycopene and therefore may be the dominant carotenoid. The highest β-carotene levels were reported for *Q. nigra* and *Q. faginea* [24]. Long-chain aliphatic alcohols were reported only for *Q. ilex* and *Q. suber* by Rabhi et al. (2016) [39]. In both species, tetracosanol was the predominant alcohol, indicating a similar alcohol profile despite species differences.

A further limitation affecting the interpretation of Table 2 is the inconsistency in how minor bioactive lipid compounds are reported across studies: concentrations are variously expressed relative to the oil (mg/kg oil or mg/100 g oil), to the dry weight of the acorn or flour (mg/kg DM), or without an explicitly stated basis. Because tocopherol, phytosterol, and carotenoid contents in acorn tissue depend strongly on total lipid content, values expressed per unit of oil cannot be directly compared with those expressed per unit of whole acorn or flour, and apparent differences between species or origins may partly reflect this reporting inconsistency rather than genuine compositional differences. Similarly, the wide range reported for total tocopherols (from approximately 30 to 4477 mg/kg oil) is difficult to interpret in the absence of harmonized units and comparable extraction and quantification methods. Standardizing the reporting basis and analytical methodology for minor lipid compounds would considerably improve the comparability of future studies and the reliability of interspecific and geographic comparisons.

## 4. Nutritional and Functional Significance of Acorn Lipids

Acorns have long been used as a food resource, particularly in Mediterranean regions, where they were consumed directly or processed into flour and oil [1,2,4,41,42]. In recent years, interest in acorns has shifted from their historical role as an alternative food source toward their potential as functional ingredients [5,21,24,43].

**Figure 1:**
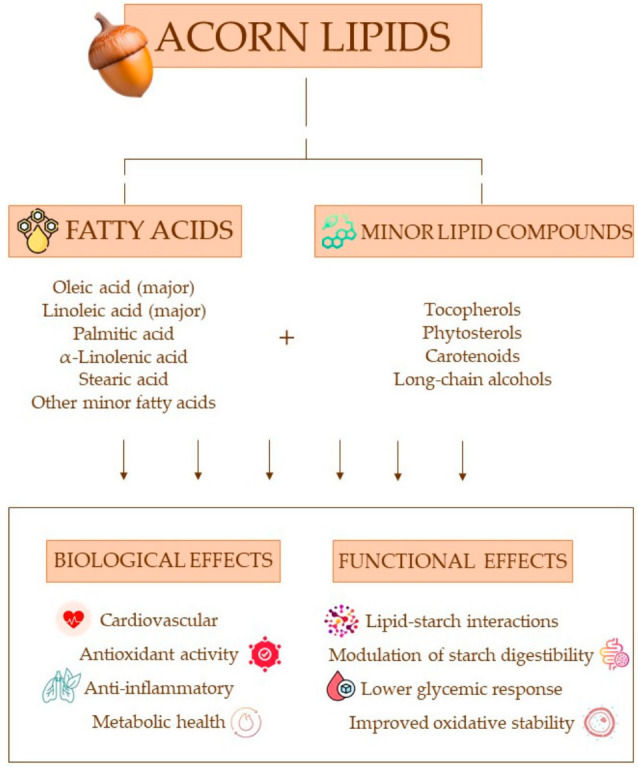
Conceptual overview of the biological and functional effects associated with acorn lipids.

The lipid fraction of acorns is characterized by the predominance of UFAs, particularly oleic acid, together with minor bioactive compounds, including tocopherols, phytosterols, carotenoids and long-chain aliphatic alcohols (Table 1 and Table 2). These constituents have been associated with antioxidant, anti-inflammatory, hypocholesterolemic and cardioprotective effects, suggesting that acorn lipids contribute to the functional value of acorn-derived foods. Beyond their nutritional relevance, lipids also influence the digestibility, oxidative stability and technological performance of acorn-derived products (Figure 1).

### 4.1. Health Implications

Acorn lipids are characterized by a favorable composition rich in oleic acid and containing several minor bioactive lipid constituents, including tocopherols, phytosterols, carotenoids and long-chain aliphatic alcohols (Table 1 and Table 2). Based on the established biological activities of these compounds in other dietary sources, acorn lipids may also possess health-promoting properties. These potential effects are thought to arise from the combined action of the major fatty acids and minor bioactive lipid constituents rather than from a single compound. However, these potential effects remain largely hypothetical because direct evidence from studies using acorn lipids is currently lacking. The predominance of monounsaturated oleic acid (18:1 n-9) in acorn lipids suggests potential cardiovascular and metabolic benefits. Consumption of oleic acid-rich foods has been associated with improved HDL-C concentrations and reduced cardiovascular risk, while experimental studies also suggest beneficial effects on blood pressure regulation and satiety [44,45,46,47,48].

As another major fatty acid of acorn lipids, linoleic acid (an omega-6 PUFA) has been associated with a lower risk of cardiovascular disease and type 2 diabetes, potentially through improvements in lipid metabolism, insulin sensitivity and anti-inflammatory mechanisms [49,50,51,52,53,54,55,56].

α-Linolenic acid is an essential omega-3 PUFA that, although present in smaller amounts, contributes to several health-beneficial effects. Appropriate intake of α-linolenic acid has been associated with cardiovascular benefits and anti-inflammatory effects, partly through its role as a precursor of longer-chain omega-3 fatty acids [57,58,59,60,61].

The tocopherols (vitamin E compounds) present in acorn lipids are lipophilic compounds that scavenge free radicals and exert antioxidant activity. They have been associated with reduced oxidative stress, anti-inflammatory effects and neuroprotection [62,63,64].

Phytosterols are plant-derived sterols primarily recognized for their cholesterol-lowering effects, thereby contributing to reduced cardiovascular risk. In addition, they have demonstrated anti-inflammatory properties, supporting their potential contribution to cardiometabolic health [65,66,67].

Carotenoids are bioactive pigments with antioxidant activity, some of which also possess provitamin A activity. They have been associated with a reduced risk of several chronic diseases through the modulation of oxidative stress and inflammation [68].

Long-chain aliphatic alcohols, particularly octacosanol and related policosanols, have been associated with cholesterol-lowering, antioxidant and anti-inflammatory activities, suggesting a potential contribution to cardiometabolic health [69,70].

It should be emphasized that the health-related evidence summarized above was not obtained from studies using acorn oil or isolated acorn lipids; rather, the potential effects have been extrapolated from research on oleic acid, linoleic acid, tocopherols, phytosterols and related compounds in other dietary sources (e.g., olive oil, sunflower oil and nuts). While this extrapolation is reasonable given the shared chemical identity of these compounds, potential matrix effects, differences in bioaccessibility and interactions with other acorn constituents may modify their physiological impact. Moreover, the few intervention studies conducted with acorn-based products used whole acorn flour rather than isolated lipid fractions, preventing attribution of the observed health effects specifically to acorn lipids [20,71]. To date, no controlled human or animal studies have evaluated acorn oil or isolated acorn lipids as the sole intervention. Therefore, the health-promoting potential of acorn lipids remains hypothetical and requires direct experimental validation.

### 4.2. Influence on Digestibility and Functional Properties

Starch is the major carbohydrate component of acorns and one of the principal determinants of their digestibility and functional properties. However, starch digestibility is also influenced by endogenous lipids naturally present in acorns, which interact with starch granules and modify their physicochemical properties and susceptibility to enzymatic hydrolysis [8,9,10,72,73]. He et al. (2022) [19] reported that acorn kernels contain approximately 5.2% fat and demonstrated that endogenous lipids interact with starch granules and influence their physicochemical properties, as well as their digestive behavior. Lipids associated with starch surfaces may increase hydrophobicity, affect water absorption and swelling during processing, and participate in the formation of starch–lipid complexes that slow down or block enzymatic hydrolysis.

The rate of starch digestion is a key determinant of the postprandial glycemic response: rapidly digested starch (RDS) is hydrolyzed quickly in the small intestine, causing a rapid increase in blood glucose concentrations. In contrast, slowly digestible starch (SDS) and resistant starch (RS) fractions delay glucose release and absorption, contributing to a lower glycemic response and improved metabolic control [73]. Consequently, food components that reduce starch digestibility are often associated with a lower glycemic index and more balanced postprandial glycemic response. He et al. (2022) [19] demonstrated that endogenous lipids, proteins and β-glucan in acorn kernels reduce starch hydrolysis through the formation of complexes with starch granules and by limiting enzyme accessibility. The presence of surface lipids increases hydrophobicity and affects starch swelling and amylose leaching, contributing to slower enzymatic digestion [74]. These mechanisms could decrease the amount of RDS and increase SDS and RS fractions while potentially attenuating postprandial glycemic excursions. Supporting this hypothesis, a clinical trial conducted by Sasani et al. (2022) [20] reported that daily consumption of acorn flour muffins for eight weeks resulted in a moderate improvement in glycated hemoglobin (HbA1c) and favorable effects on appetite regulation in patients with type 2 diabetes. Nevertheless, the observed benefits cannot be attributed solely to endogenous lipids. Acorn flour is also rich in dietary fiber, polyphenols, and other bioactive compounds that may independently reduce starch digestion, inhibit carbohydrate-hydrolyzing enzymes and contribute to its generally low glycemic index [75]. Therefore, the glycemic effects of acorn-based foods are likely attributable to multiple food components, rather than solely to starch–lipid interactions.

Besides their direct influence on starch digestibility, acorn lipids may also participate in interactions with other food constituents that influence the physicochemical properties, stability and nutrient bioaccessibility of acorn-based foods. He et al. (2022) [19] suggested that lipids participate in interactions between starch and proteins and act as a molecular bridge contributing to the formation of complex food structures. In addition, interactions between acorn lipids and phenolic compounds may influence nutrient stability and bioaccessibility, although direct evidence for these interactions remains limited [11,76]. Antioxidant constituents present in acorns, including lipid-associated tocopherols and phenolic compounds, may also help protect acorn UFAs from oxidative degradation and thereby contribute to product stability during processing and storage [11]. However, direct investigations of lipid–protein and lipid–polyphenol interactions in acorn-enriched foods remain limited. Therefore, the contribution of acorn lipids to nutrient bioaccessibility and functional properties has not yet been fully investigated and requires further research [11,19,21,76].

## 5. Oxidative Stability and Technological Applications

**Figure 2:**
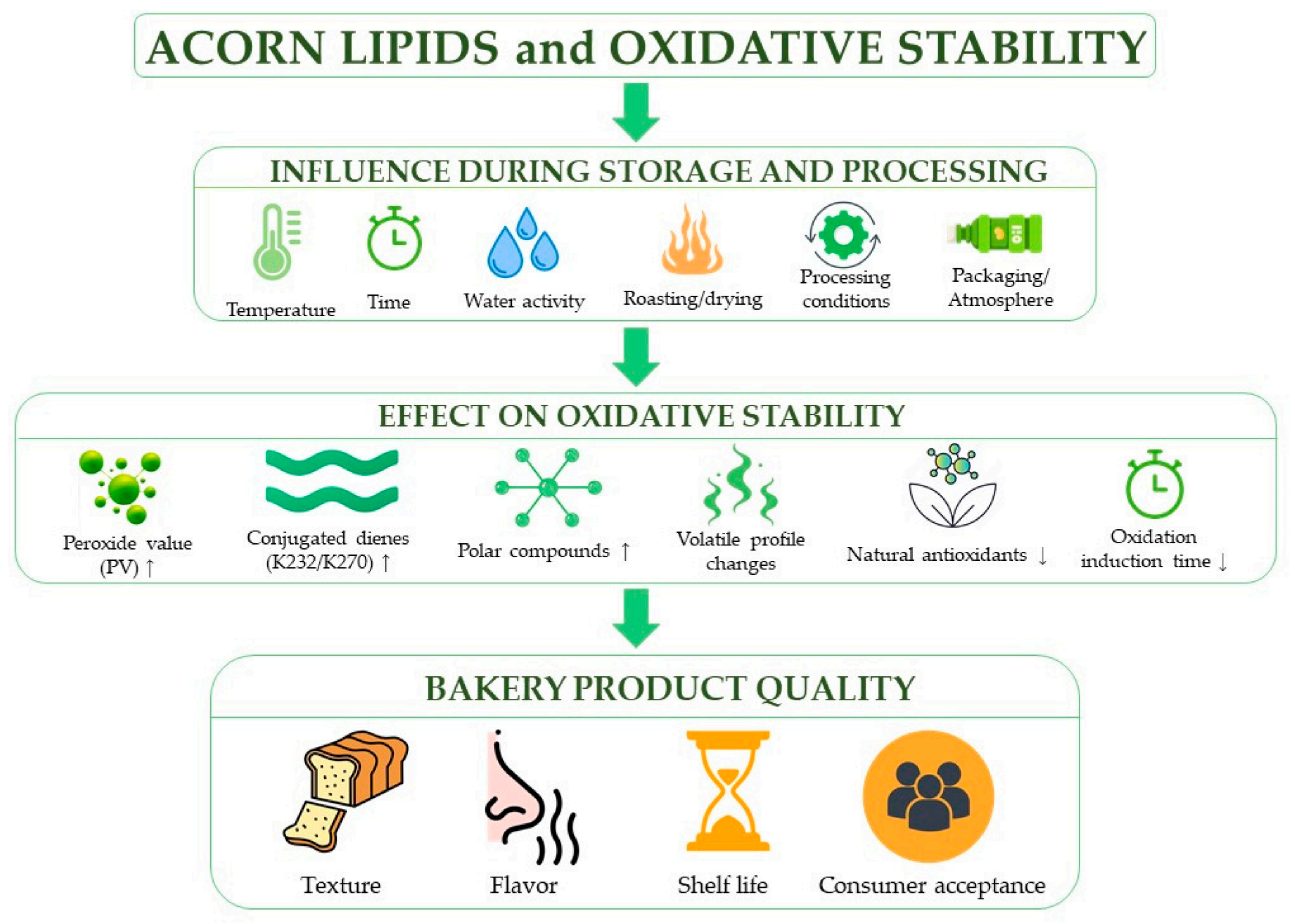
Conceptual overview of factors affecting the oxidative stability of acorn lipids and their impact on bakery product quality. Upward (↑) and downward (↓) arrows indicate the increase or decrease of specific oxidative markers and stability parameters.

Oxidative stability is an important quality parameter of lipid-containing foods, defined as the resistance of lipids to oxidative deterioration during processing and storage. Lipid oxidation leads to the formation of primary and secondary oxidation products that negatively affect nutritional quality, sensory characteristics and shelf life. In addition, lipid peroxidation products have been associated with oxidative stress following the consumption of lipid-rich meals. Therefore, lipid oxidation is recognized as a major cause of quality deterioration in food products [77,78]. The susceptibility of a lipid fraction to oxidation is mostly influenced by its fatty acid composition—namely, the degree of unsaturation—and the presence of naturally occurring antioxidants such as phenolic compounds and tocopherols (Figure 2) [79].

As previously stated, acorn lipids contain a high proportion of UFAs, mainly oleic and linoleic acids, which provide desirable nutritional properties but may also increase susceptibility to oxidative degradation. Nevertheless, studies investigating the storage stability of acorn oils have demonstrated limited oxidative deterioration under the tested conditions. Makhlouf et al. (2021) [14] observed that although peroxide values (PV), polar compounds and other oxidation markers increased during storage, PV remained between 1.46 and 1.94 mEq O_2_/kg oil after 180 days of storage in the dark at room temperature. The authors reported that this behavior may be partly explained by the presence of antioxidant compounds naturally occurring in acorns.

Acorn lipids also contribute to the technological properties of food products. Acorn-based ingredients have been incorporated into a wide range of food products, including bread, biscuits, cakes, muffins, pasta and pita bread, contributing to improved nutritional quality, enhanced antioxidant capacity and modified rheological and textural properties [12,21,33,80,81,82,83,84,85,86,87,88,89,90]. Furthermore, acorn oil has been successfully incorporated into oleogel-based chocolate spreads where it improved physical stability of the spread and demonstrated the potential of acorn lipids as functional ingredients in innovative food formulations [91].

### 5.1. Oxidative Stability of Acorn Lipids

Acorn oils are characterized by a predominance of oleic acid (approximately 60–65%), followed by linoleic acid and lower proportions of SFAs, which make their fatty acid profile similar to that of olive oil. Acorn oils also contain considerable amounts of tocopherols, phytosterols and phenolic compounds that contribute to their antioxidant potential. Although their high UFA content may increase susceptibility to oxidative degradation, acorn oils have demonstrated measurable resistance to oxidation under specific experimental conditions. Lopes and Bernardo-Gil (2005) [13] reported Rancimat induction times of 16 and 78 h for SFE-CO_2_- and n-hexane-extracted *Q. rotundifolia* oils, respectively, and 60 h for n-hexane-extracted *Q. suber* oil. More recent investigations have also shown that oxidative stability varies among *Quercus* species, indicating that botanical origin and differences in chemical composition may influence the oxidative behavior of acorn oils. Hence, the stability of acorn lipids results from the combined effects of fatty acid composition and minor lipid compounds with antioxidant activities [14]. The predominance of oleic acid over more oxidation-prone polyunsaturated fatty acids, together with endogenous antioxidants such as tocopherols and phenolic compounds, likely delays lipid peroxidation by reducing radical propagation and scavenging reactive oxygen species.

The oxidative stability of acorn oils has been evaluated using both conventional physicochemical quality indices and accelerated oxidation assays, including peroxide value (PV), ultraviolet absorbance (K_232_/K_270_), Rancimat and RapidOxy induction times, pressure differential scanning calorimetry (PDSC) oxidation onset and maximum oxidation times, polar compounds, and volatile oxidation products [13,14,15,17,32,91]. Across different *Quercus* species, saponification values generally range from 160.3 to 219.4 mg KOH/g oil, while iodine values vary between 75.2 and 129.6 g I_2_/100 g oil, which reflects differences in the degree of unsaturation. Acidity values are generally low, although they vary considerably depending on species, fruit quality and extraction method, ranging from 0.43 to 5.8 mg KOH/g oil (or equivalent free fatty acid values). Similarly, PVs remain low in freshly extracted oils, typically ranging from 0.20 to 2.89 mEq O_2_/kg oil. These low PVs indicate limited primary lipid oxidation at the time of analysis. Refractive indices reported for acorn oils are remarkably consistent (1.449–1.470) and comparable to those of olive oil, further supporting the favorable physicochemical characteristics of acorn oils. Nevertheless, these quality indices are influenced by species, geographical origin, storage conditions, fruit maturity, and extraction technique [13,14,36,92,93,94].

During storage, acorn oils undergo gradual oxidative changes that are characteristic of vegetable oils. The magnitude of these changes depends on the species, extraction method and storage conditions, as summarized in Table 3. Makhlouf et al. (2021) [14] investigated the stability of oils obtained from *Q. ilex*, *Q. suber* and *Q. coccifera* during 180 days of storage in the dark at room temperature. As expected, oxidation indices, including PV and specific UV absorbance (K_232_ and K_270_), increased throughout storage. These changes indicate the progressive formation of primary and secondary lipid oxidation products. At the same time, the concentrations of natural antioxidants, particularly phenolic compounds, declined, while antioxidant activity and oxidative induction time also decreased. These changes can be explained by the gradual depletion of endogenous antioxidant compounds that initially delayed lipid oxidation. In parallel, the total content of polar compounds increased because of the accumulation of free fatty acids, diacylglycerols and oxidized triacylglycerols. The volatile profiles also changed because of the loss of most volatile compounds during storage. No volatile oxidation markers were detected, except for hexanal in *Q. coccifera*, probably because of the mild storage conditions. Volatile aldehydes, ketones and related secondary oxidation products are important indicators of advanced lipid oxidation because they contribute to undesirable sensory characteristics. Furthermore, when present at elevated concentrations, these compounds have been associated with adverse biological effects due to their high chemical reactivity [95,96]. Despite these oxidative changes, PVs remained between 1.46 and 1.94 mEq O_2_/kg oil after 180 days of storage. Among the investigated species, *Q. coccifera* exhibited the longest oxidative induction times, whereas *Q. ilex* showed the smallest increases in PV and UV absorbance during storage. In contrast, *Q. suber* experienced the greatest deterioration, with the largest increases in oxidation markers and polar compounds and the greatest reduction in induction time (Table 3) [14]. Similarly, Kowalska et al. (2026) [17] reported species- and extraction-dependent differences in oxidative stability, with *Q. robur* oils exhibiting substantially longer PDSC oxidation times than the corresponding *Q. rubra* oils, while mechanically pressed oils showed the highest peroxide values and the shortest oxidation times (Table 3). These findings are consistent with earlier observations that acorn oils can resist oxidation under the tested conditions and suggest that differences in oxidative stability are influenced by species, extraction technique, and the content of naturally occurring antioxidant compounds [13].

Processing conditions may influence the oxidative stability of acorn lipids by altering the content of naturally occurring antioxidant compounds. Thermal treatments (drying, roasting, etc.) are generally expected to affect phenolic compounds and tocopherols and consequently influence the resistance of lipids to oxidation. However, the available evidence on acorn lipids is limited. Rakić (2000) [32] reported that extracts obtained from both dried and heat-treated acorns exhibited antioxidant activity, while heat treatment (210 °C for 15 min) did not reduce their antioxidative properties—in fact, it resulted in greater antioxidant activity than that of untreated acorns. Furthermore, Kowalska et al. (2026) [17] demonstrated that the extraction method also influences oil quality: mechanically pressed oils exhibited higher acid values and PVs than solvent-extracted oils. Likewise, Lopes and Bernardo-Gil (2005) [13] reported that *Q. rotundifolia* oil extracted with n-hexane exhibited greater oxidative stability during storage than the corresponding supercritical CO_2_ extract under the investigated conditions (Table 3). Nevertheless, species differences had a greater effect on oxidative stability than the extraction technique itself, as previously stated. Overall, the effects of processing on acorn lipid stability remain insufficiently investigated and require further research.

### 5.2. Application in Bakery and Other Food Products

Although the technological properties of acorn-enriched bakery products result from interactions between starch, proteins, dietary fiber and phenolic compounds, the lipid fraction also contributes to dough rheology, crumb structure and product stability. However, the specific contribution of acorn lipids is difficult to isolate because most food-application studies have used whole acorn flour rather than purified acorn oil or individual lipid fractions. Consequently, findings from flour-enriched products should be interpreted as evidence concerning acorn lipids within a complex food matrix, not as direct evidence of lipid functionality.

The incorporation of acorn-derived ingredients into bakery products has attracted increasing attention because of their nutritional composition and functional properties. Acorn flour contains unsaturated lipids, dietary fiber, starch and phenolic compounds. As a lipid-containing ingredient, it has been investigated in bread, gluten-free bakery products, biscuits, cakes, muffins and other foods [12,21,33,80,81,82,83,84,85,86,87,88,89,90]. In these formulations, the lipid fraction acts together with starch, proteins, fiber and phenolic compounds; therefore, observed changes in dough behavior, texture and oxidative stability cannot be attributed exclusively to acorn lipids.

Bread is the acorn-enriched product studied most extensively. Studies have shown that partial substitution of wheat flour with acorn flour improves the nutritional quality of bread by increasing dietary fiber, mineral content, UFAs and antioxidant capacity. At the same time, incorporation of acorn flour affects dough rheology: the lack of gluten in acorn flour and the presence of lipids and fiber modify water absorption, dough development and gas retention. Moderate substitution levels generally produce acceptable bread quality, while higher substitution levels tend to reduce loaf volume and increase crumb firmness due to dilution of the gluten network. Although endogenous acorn lipids may participate in starch–lipid and protein–lipid interactions during mixing and baking, the available bread studies do not experimentally distinguish their effects from those of the other flour constituents [12,21,97,98,99]. 

A similar limitation applies to gluten-free bread. Acorn flour has been used to increase the contents of UFAs, fiber, minerals and antioxidant compounds, while moderate incorporation levels generally maintain acceptable technological and sensory properties [80,81,82,83,84]. However, because these studies evaluated whole flour, their results primarily demonstrate the application potential of a lipid-containing acorn ingredient rather than the independent technological functionality of its lipid fraction.

In biscuits, cookies, cakes and muffins, acorn flour has similarly improved the nutritional profile and antioxidant capacity, whereas increasing substitution levels have generally produced darker, denser or firmer products [43,71,85,86,87,88]. These applications broaden the range of foods through which acorn lipids may be incorporated, but they provide little direct information about the behavior of the lipids themselves during processing or storage.

Beyond bakery products, acorn-derived ingredients have also been incorporated into pasta, milk puddings, beverages, beer and coffee substitutes [75,89,100,101]. Among the available applications, the clearest example involving isolated acorn lipids is the incorporation of acorn oil into oleogel-based chocolate spreads. The acorn-oil oleogel contributed to the physical stability and structural properties of the spread, demonstrating the potential of acorn oil as a functional lipid phase rather than only as a component of whole acorn flour [91]. Oleogelation may consequently offer a strategy for incorporating the UFA-rich acorn lipid fraction into structured foods while reducing reliance on conventional solid fats rich in SFAs.

Despite their promising applications, some limitations should also be considered when incorporating acorn-derived ingredients into foods. Acorns naturally contain antinutritional compounds, particularly tannins, which may reduce protein digestibility and mineral bioavailability and may cause bitterness. However, traditional processing methods such as soaking, leaching and thermal treatment substantially reduce tannin content and improve both nutritional quality and sensory acceptability [24,30,31]. In addition, although allergic reactions to acorns appear to be uncommon, the allergenic potential of acorn-derived foods has not been extensively investigated and should be considered in future safety assessments [102,103]. From a lipid-specific perspective, future studies should investigate the behaviour of extracted acorn oil during food processing and storage, including its effects on rheology, texture, sensory properties and oxidative stability during shelf life. Direct comparisons between isolated acorn oil, conventional edible oils and whole acorn flour are particularly necessary to distinguish the functionality of the lipid fraction from that of the complete acorn matrix.

## 6. Research Gaps and Future Perspectives

Although the lipid fraction of acorns has been characterized in increasing detail over the past two decades, a critical synthesis of the available literature reveals several methodological and conceptual gaps that currently limit both scientific interpretation and practical application of this knowledge. These gaps are summarized below and should guide the design of future studies on acorn lipids.

(1) Confounded sources of compositional variability. Reported differences in lipid content and fatty acid profiles among *Quercus* species are consistently attributed to genetic, environmental, and methodological factors, yet almost no studies have been designed to statistically separate these sources of variation, for example, through common-garden trials, multi-year sampling of the same population, or standardized interlaboratory comparisons. Until such designs are applied, claims about “species-specific” lipid profiles should be interpreted with caution.
(2) Lack of standardized reporting units. Total lipid content, fatty acid composition, and minor bioactive compound concentrations are expressed on inconsistent bases (fresh weight, dry weight, flour weight, or oil weight) across studies, which precludes direct quantitative comparison and meta-analysis. Adoption of a common reporting standard, analogous to those used for other vegetable oils, would substantially improve the comparability of future research.
(3) Whole-flour versus isolated-lipid confound. The vast majority of studies on the nutritional, technological, and health-related properties of acorn-derived foods have used whole acorn flour, in which lipids coexist with starch, dietary fiber, protein, and phenolic compounds. Consequently, the specific contribution of the lipid fraction to observed effects on starch digestibility, dough rheology, or clinical outcomes cannot be isolated from the existing evidence. Studies using extracted acorn oil or defatted acorn flour as parallel treatments would help resolve this confound.
(4) Scarcity of direct evidence for health effects. As discussed in Section 4.1, the proposed health benefits of acorn lipids rely heavily on evidence generated for the same compounds in other food matrices rather than on acorn-specific intervention studies. Controlled trials using acorn oil or isolated acorn lipids as the primary intervention are needed to substantiate these health claims.
(5) Limited data on processing and storage effects. Only a few studies have examined how common processing operations, such as drying, roasting, dehulling, or mechanical versus solvent extraction, affect the retention of tocopherols, phytosterols, and other antioxidants and, consequently, the oxidative stability of acorn lipids during storage and food processing. Systematic evaluation of processing parameters would support the technological optimization of products containing acorn lipids.
(6) Underexplored mechanistic interactions. While starch–lipid interactions and their influence on digestibility have begun to be investigated, the mechanisms by which acorn lipids interact with proteins and phenolic compounds, as well as the effects of these interactions on nutrient bioaccessibility and product functionality, remain largely unexplored and warrant dedicated mechanistic studies.

## 7. Conclusions

Acorn lipids represent a valuable but still underutilized component of acorns and acorn-derived foods. Across different *Quercus* species, the lipid fraction is generally characterized by high proportions of UFAs, particularly oleic acid and, to a lesser extent, linoleic acid. Although the total lipid content varies among species and populations, these fatty acids consistently represent the major components of acorn oils. This composition is similar to that of other highly valued plant oils, such as olive oil, and contributes to the potential health-promoting properties of acorn lipids.

Furthermore, acorn lipids contain several minor bioactive compounds, including tocopherols, phytosterols, carotenoids and long-chain aliphatic alcohols. These compounds have been associated with antioxidant, anti-inflammatory, hypocholesterolemic and cardioprotective properties, suggesting that the health-promoting potential of acorns may arise from both fatty acids and minor lipid compounds. Consequently, acorn lipids represent a promising source of bioactive compounds, supporting their potential use in the development of functional foods.

The available evidence also indicates that acorn lipids influence food functionality: they are involved in starch–lipid interactions that affect starch digestibility and glycemic response. Despite their high content of UFAs, acorn oils showed limited oxidative deterioration under the storage conditions evaluated. Studies investigating storage conditions have shown that oxidative deterioration develops gradually, although direct comparisons with conventional vegetable oils remain limited by differences in analytical methods and experimental conditions.

Despite their potential, the presence of antinutritional compounds and the limited information regarding the allergenic potential of acorns should also be considered when developing acorn-derived food products.

Acorn-derived ingredients have potential for use in bread, gluten-free products, biscuits, cakes, muffins, pasta and other food applications because they improve nutritional value and antioxidant capacity and contribute to product functionality. However, most available studies have examined whole acorn flour instead of the isolated lipid fraction, making it difficult to determine the specific contribution of lipids to the occurring effects. In addition, considerable variability exists among *Quercus* species and analytical methods, which complicates direct comparisons across studies.

Overall, the current literature highlights the substantial nutritional and technological potential of acorn lipids. However, the key research priorities required to close these gaps and enable the effective utilization of acorn lipids in future food and nutrition applications are outlined in Section 6.

## Figures and Tables

**Table 1:** Total lipid content and fatty acid composition of acorns from selected *Quercus* species.

Species	Geographic Origin	Matrix Analysed	Basis	Total Lipid Content (%)	Dominant Fatty Acids (%)				Reference
					Oleic Acid	Linoleic Acid	Palmitic Acid	Other Notable Fatty Acids	
*Q. canariensis*	Tunisia (Ghardimaou province)	Acorn flour	Dry weight (DW)	4.73	56.84	23.68	14.76	Stearic (1.48); α-Linolenic (1.46)	[18]
*Q. faginea*	Portugal (Trás-os-Montes region)	Acorn kernels	DW	3.82	59.01	21.56	14.62	Stearic (1.77); Eicosenoic (1.12)	[29]
*Q. faginea*	Portugal (Trás-os-Montes region)	Acorn kernel	DW	2.0	51.0	20.0	18.0	α-Linolenic (5.0); Stearic (2.0)	[24]
*Q. frainetto*	Turkey	Ground acorn kernels	FW (air-dried sample)	1.17	25.7	38.7	20.9	α-linolenic (4.4); Stearic (3.2)	[16]
*Q. hartwissiana*	Turkey	Ground acorn kernels	FW (air-dried sample)	1.01	21.4	49.1	17.1	α-Linolenic (5.3); Stearic (1.8)	[16]
*Q. ilex*	Turkey	Ground acorn kernels	FW (air-dried sample)	0.80	15.3	31.9	27.7	α-Linolenic (8.5); Stearic (4.4)	[16]
*Q. ilex*	Portugal (Trás-os-Montes region)	Acorn kernels	DW	12.01	69.05	13.34	12.60	Elaidic (3.02); Eicosenoic (0.59)	[29]
*Q. ilex*	Portugal (Montemor-o-Novo)	Acorn flour	DW	13.41	59.85	15.49	14.98	Stearic (3.25); Vaccenic (0.82)	[31]
*Q. ilex*	Tunisia (Queslatia province)	Acorn flour	DW	9.32	63.96	15.41	15.10	Stearic (3.27); α-Linolenic (0.74)	[18]
*Q. ilex*	Portugal (Trás-os-Montes region)	Acorn kernels	DW	8.0	64.0	14.0	14.0	α-Linolenic (2); Stearic (1.0)	[24]
*Q. nigra*	Portugal (Trás-os-Montes region)	Acorn kernels	DW	2.54	47.12	31.21	15.24	Eicosenoic (2.43); Stearic (1.64)	[29]
*Q. nigra*	Portugal (Trás-os-Montes region)	Acorn kernels	DW	2.0	48.0	26.0	17.0	α-Linolenic (4.0); Stearic (2.0)	[24]
*Q. petraea*	Turkey	Ground acorn kernels	FW (air-dried sample)	2.75	41.4	31.1	19.6	α-Linolenic (2.1); Stearic (1.8)	[16]
*Q. pontica*	Turkey	Ground acorn kernels	FW (air-dried sample)	1.59	32.6	35.9	18.2	α-Linolenic (5.5); Stearic (2.1)	[16]
*Q. pubescens*	Turkey	Ground acorn kernels	FW (air-dried sample)	1.16	16.9	38.7	22.8	α-Linolenic (6.1); Stearic (3.1)	[16]
*Q. pyrenaica*	Portugal (Castelo Branco district)	Acorn flour	DW	7.19	57.08	24.74	14.01	Stearic (1.81); Palmitoleic (0.4)	[30]
*Q. robur*	Poland (Krotoszyn area)	Ground acorn kernels	DW	7.01	52.08	30.26	13.09	α-Linolenic (1.34); Stearic (1.89)	[17]
*Q. robur*	Portugal (Porto district)	Acorn flour	DW	6.08	45.90	33.05	15.93	Stearic (1.72); α-Linolenic (0.45)	[30]
*Q. robur*	Turkey	Ground acorn kernels	FW (air-dried sample)	0.99	22.6	41.9	18.9	α-Linolenic (4.0); Stearic (2.0)	[16]
*Q. rotundifolia*	Portugal (Montemor-o-Novo)	Acorn flour	DW	8.44	60.92	15.91	14.21	Stearic (2.33); Vaccenic (0.93)	[31]
*Q. rotundifolia*	Spain (Badajoz province)	Acorn flour	DW	11.27	69.37	17.82	10.33	Stearic (1.44); α-Linolenic (0.27)	[30]
*Q. rotundifolia*	Portugal (Évora and Portalegre region)	Acorn flour	Fresh weight (FW)	11.39	66.26	14.72	14.11	Stearic (3.68); α-Linolenic (0.51)	[5]
*Q. rotundifolia*	Portugal (Alto Alentejo region)	Ground acorn kernels	FW (air-dried sample)	12.1	64.81	16.43	13.42	Stearic (2.50); α-Linolenic (0.57)	[13]
*Q. rubra*	Poland (Krotosyzn area)	Ground acorn kernels	DW	17.56	64.22	22.19	9.85	Stearic (2.12); Eicosenoic (0.61)	[17]
*Q. suber*	Tunisia (Ain Drahem province)	Acorn flour	DW	5.47	65.28	17.44	13.24	α-Linolenic (1.49); Stearic (1.20)	[18]
*Q. suber*	Portugal (Trás-os-Montes region)	Acorn kernels	DW	3.86	54.59	23.19	14.71	Eicosenoic (3.69); Stearic (1.49)	[29]
*Q. suber*	Portugal (Trás-os-Montes region)	Acorn kernels	DW	3.0	55.0	21.0	14.0	α-Linolenic (4.0); Stearic (2.0)	[24]
*Q. suber*	Portugal (Alto Alentejo region)	Ground acorn kernels	FW (air-dried sample)	6.0	66.44	17.03	12.08	Stearic (1.34); α-Linolenic (1.00)	[13]
*Q. vulcanica*	Turkey	Ground acorn kernels	FW (air-dried sample)	0.75	50.2	25.1	13.8	Stearic (4.0); α-Linolenic (2.6)	[16]

Lipid contents are reported on the basis described in the original publications. Differences among studies may also reflect variations in extraction method, analytical protocol, sample pretreatment, maturity stage, harvest season, and other experimental conditions. Therefore, the reported values should be interpreted as indicative and should not be directly compared across studies.

**Table 2:** Occurrence of tocopherols, phytosterols, carotenoids and other minor bioactive lipid compounds in acorns of selected *Quercus* species.

Compound Class	Dominant Compounds	Species	Main Finding	Reference
Tocopherols	α-Tocopherol	*Q. canariensis*	α-Tocopherol: 14.03–57.67 mg/kg oil	[15]
Tocopherols	α-, γ-, δ-Tocopherol (γ predominant)	*Q. coccifera*	Total tocopherols: 32.54 mg/kg oil	[38]
Tocopherols	α-, γ-, δ-Tocopherol (γ predominant)	*Q. faginea*	Total tocopherols: 31.83 mg/kg oil	[38]
Tocopherols	α-, γ-, δ-Tocopherol (γ predominant)	*Q. ilex*	Total tocopherols: 45.25 mg/kg oil	[38]
Tocopherols	α-, γ-, δ-Tocopherol (γ predominant)	*Q. pyrenaica*	Total tocopherols: 32.29 mg/kg oil	[38]
Tocopherols	α-, γ-, δ-Tocopherol (γ predominant)	*Q. suber*	Total tocopherols: 31.60 mg/kg oil	[38]
Tocopherols	α-, β, γ- and δ-Tocopherol (α predominant)	*Q. faginea*	Total vitamin E: 130 mg/kg oil	[24]
Tocopherols	α-, β, γ- and δ-Tocopherol (γ predominant)	*Q. ilex*	Total vitamin E: 60 mg/kg oil	[24]
Tocopherols	α-, β, γ- and δ-Tocopherol (γ predominant)	*Q. nigra*	Total vitamin E: 100 mg/kg oil	[24]
Tocopherols	α-, β, γ- and δ-Tocopherol (γ predominant)	*Q. suber*	Total vitamin E: 70 mg/kg oil	[24]
Tocopherols	α- and γ-Tocopherol (γ predominant)	*Q. ilex*	Total tocopherols: 190.25 mg/kg oil	[39]
Tocopherols	α- and γ-Tocopherol (γ predominant)	*Q. suber*	Total tocopherols: 141.15 mg/kg oil	[39]
Tocopherols	α- and γ-Tocopherol (γ predominant)	*Q. rotundifolia*	γ-Tocopherol: 57.5–107.9 mg/kg DM	[27]
Tocopherols	α- and γ + δ-Tocopherol (γ predominant)	*Q. rotundifolia*	Total tocopherols: 1006 mg/kg oil	[13]
Tocopherols	α- and γ + δ-Tocopherol (γ predominant)	*Q. suber*	Total tocopherols: 1486 mg/kg oil	[13]
Tocopherols	α-, β, γ- and δ-Tocopherol (γ predominant)	*Q. robur*	Total tocopherols: 4477 mg/kg oil	[28]
Tocopherols	α-, β, γ- and δ-Tocopherol (β predominant)	*Q. rubra*	Total tocopherols: 784 mg/kg oil	[28]
Phytosterols	β-Sitosterol, campesterol, stigmasterol,	*Q. coccifera*	β-Sitosterol: 93.77% of total sterols	[40]
Phytosterols	β-Sitosterol, campesterol, stigmasterol,	*Q. ilex*	β-Sitosterol: 91.31% of total sterols	[40]
Phytosterols	β-Sitosterol, campesterol, stigmasterol, (β-Sitosterol predominant)	*Q. ilex*	Total sterols: 8714.92 mg/kg oil	[39]
Phytosterols	β-Sitosterol, campesterol, stigmasterol, (β-Sitosterol predominant)	*Q. suber*	Total sterols: 8797.92 mg/kg oil	[39]
Phytosterols	β-Sitosterol, campesterol, stigmasterol, cholesterol (β-Sitosterol predominant)	*Q. rotundifolia*	Total sterols: 4478 mg/kg oil	[13]
Phytosterols	β-Sitosterol, campesterol, stigmasterol, cholesterol (β-Sitosterol predominant)	*Q. suber*	Total sterols: 4764 mg/kg oil	[13]
Phytosterols	β-Sitosterol, campesterol, stigmasterol (β-Sitosterol predominant)	*Q. robur*	Total sterols: 2449 mg/kg oil	[28]
Phytosterols	β-Sitosterol, campesterol, stigmasterol (β-Sitosterol predominant)	*Q. rubra*	Total sterols: 2714 mg/kg oil	[28]
Carotenoids	β-Carotene, lycopene	*Q. faginea*	β-Carotene: 1312 mg/kg DM	[24]
Carotenoids	β-Carotene, lycopene	*Q. ilex*	β-Carotene: 473 mg/kg DM	[24]
Carotenoids	β-Carotene, lycopene	*Q. nigra*	β-Carotene: 1421 mg/kg DM	[24]
Carotenoids	β-Carotene, lycopene	*Q. suber*	β-Carotene: 1301 mg/kg DM	[24]
Long-chain aliphatic alcohols	Docosanol, tetracosanol, hexacosanol, octacosanol (tetracosanol predominant)	*Q. ilex*	Total aliphatic alcohols: 2198.81 mg/kg oil	[39]
Long-chain aliphatic alcohols	Docosanol, tetracosanol, hexacosanol, octacosanol (tetracosanol predominant)	*Q. suber*	Total aliphatic alcohols: 2248.87 mg/kg oil	[39]

DM—dry matter.

**Table 3:** Comparative oxidative stability of acorn oils reported in the literature.

Species/Oil Extraction Technique	Method	Key Results	Main Conclusion	Reference
*Q. rotundifolia*/SFE-CO_2_ (18 MPa, 313 K)	PV Rancimat (110 °C) Storage at 60 °C, light + air	Initial PV: 7 mEq O_2_/kg IT: 16 h PV after 19 d: ≈74 mEq O_2_/kg	Lower stability than the n-hexane-extracted *Q. rotundifolia* oil under the conditions of this study	[13]
*Q. rotundifolia*/n-hexane (Soxhlet)	PV Rancimat (110 °C) Storage at 20 °C under different light/air conditions Storage at 60 °C, light + air	Initial PV: 5 mEq O_2_/kg IT: 78 h PV after 19 d at 60 °C: ≈36 mEq O_2_/kg	Greater stability than the corresponding SFE-CO_2_ oil under the conditions of this study; oxygen exposure promoted oxidation more strongly than light	[13]
*Q. suber*/n-hexane (Soxhlet)	PV Rancimat (110 °C) Storage at 60 °C, light + air	Initial PV: 6 mEq O_2_/kg IT: 60 h PV after 22 d: ≈20 mEq O_2_/kg	Oxidative behaviour did not differ significantly from n-hexane-extracted *Q. rotundifolia* oil during storage under the conditions of this study	[13]
*Q. ilex*/hexane (Soxhlet)	Storage: 180 d, dark, 23 ± 2 °C PV, K_232_/K_270_ RapidOxy (140 °C, 700 kPa) Polar compounds	PV at T_180_: 1.94 mEq O_2_/kg (+29%) K_232_ at T_180_: 1.56 (+1%); K_270_: 0.50 (+2%) IT: 215.63 → 166.95 min (−22.5%) Polar compounds: 2.39 → 3.34%	Smallest changes in PV, UV indices and IT during storage within the study	[14]
*Q. suber*/hexane (Soxhlet)	Storage: 180 d, dark, 23 ± 2 °C PV, K_232_/K_270_ RapidOxy (140 °C, 700 kPa) Polar compounds	PV at T_180_: 1.46 mEq O_2_/kg (+76%) K_232_ at T_180_: 2.00 (+23%); K_270_: 0.66 (+29%) IT: 284.04 → 195.88 min (−31.0%) Polar compounds: 3.24 → 5.36% (≈+65%)	Greatest overall storage-related deterioration within the study	[14]
*Q. coccifera*/hexane (Soxhlet)	Storage: 180 d, dark, 23 ± 2 °C PV, K_232_/K_270_ RapidOxy (140 °C, 700 kPa) Polar compounds	PV at T_180_: 1.68 mEq O_2_/kg (+68%) K_232_ at T_180_: 2.59 (+8%); K_270_: 1.34 (+18%) IT: 381.24 → 265.58 min (−30.3%) Polar compounds: 2.13 → 3.01% (≈+41%)	Longest induction time both before and after storage within the study	[14]
*Q. rubra*/hexane (Soxhlet)	PV PDSC (120 °C; O_2_: 1350–1400 kPa)	PV: 1.10 ± 0.08 mEq O_2_/kg τon: 126.11 ± 1.22 min τmax: 136.38 ± 1.43 min	Highest PDSC stability among the *Q. rubra* oils evaluated in this study	[17]
*Q. rubra*/hexane (cold extraction)	PV PDSC (120 °C; O_2_: 1350–1400 kPa)	PV: 1.23 ± 0.10 mEq O_2_/kg τon: 106.29 ± 1.11 min τmax: 117.12 ± 1.16 min	Intermediate PDSC stability among the *Q. rubra* oils evaluated in this study	[17]
*Q. rubra*/mechanical pressing	PV PDSC (120 °C; O_2_: 1350–1400 kPa)	PV: 2.89 ± 0.10 mEq O_2_/kg τon: 99.78 ± 0.67 min τmax: 110.44 ± 0.87 min	Highest PV and shortest PDSC oxidation times among the *Q. rubra* oils evaluated in this study	[17]
*Q. robur*/hexane (Soxhlet)	PV PDSC (120 °C; O_2_: 1350–1400 kPa)	PV: 2.78 ± 0.12 mEq O_2_/kg τon: 212.95 ± 2.56 min τmax: 227.92 ± 2.43 min	Longer PDSC oxidation times than cold-extracted *Q. robur* oil and corresponding *Q. rubra* oil	[17]
*Q. robur*/hexane (cold extraction)	PV PDSC (120 °C; O_2_: 1350–1400 kPa)	PV: 2.37 ± 0.08 mEq O_2_/kg τon: 149.41 ± 1.25 min τmax: 156.04 ± 0.86 min	Lower PDSC stability than Soxhlet-extracted *Q. robur* oil, but higher than cold-extracted *Q. rubra* oil	[17]

Values preceded by ≈ were estimated from the published figures.

## Data Availability

No new data were created or analyzed in this study. Data sharing is not applicable to this article.

## Abbreviations

The following abbreviations are used in this manuscript:

DW	Dry weight
FW	Fresh weight
HDL-C	High-density lipoprotein cholesterol
IT	Induction time
LDL-C	Low-density lipoprotein cholesterol
MUFA	Monounsaturated fatty acid(s)
PUFA	Polyunsaturated fatty acid(s)
PV	Peroxide value
RDS	Rapidly digestible starch
RS	Resistant starch
SDS	Slowly digestible starch
SFA	Saturated fatty acid(s)
UFA	Unsaturated fatty acid(s)
UV	Ultraviolet
